# Innate Lymphoid Cells in Renal Inflammation

**DOI:** 10.3389/fimmu.2020.00072

**Published:** 2020-01-29

**Authors:** Martina Becker, Ann-Christin Gnirck, Jan-Eric Turner

**Affiliations:** III Department of Medicine and Hamburg Center for Translational Immunology, University Medical Center Hamburg-Eppendorf, Hamburg, Germany

**Keywords:** innate lymphoid cells, chronic kidney disease, acute kidney injury, glomerulonephritis, ILC modulation

## Abstract

Since their identification as a separate family of leukocytes, Innate lymphoid cells (ILCs) have been shown to play crucial roles in immune-mediated diseases and repair mechanisms that restore tissue integrity after injury. ILCs mainly populate non-lymphoid tissues where they form intricate circuits with parenchymal cells to regulate tissue immunity and organ homeostasis. However, the specific phenotype and function of ILC populations that reside in specific anatomical locations, such as the kidney, still remains poorly understood. In this review, we discuss tissue-specific properties of kidney-residing ILCs and summarize recent advances in the understanding of ILC biology in kidney diseases that might pave the way for development of novel treatment strategies in humans.

## Introduction

Chronic kidney disease (CKD) affects ~10% of the population in industrialized countries and is a major risk factor for cardiovascular mortality (1). CKD often shows a progressive course leading to end stage renal disease with the need for renal replacement therapy (dialysis or kidney transplantation), resulting in substantial morbidity and mortality of affected patients. Diabetes mellitus and arterial hypertension are the most common diseases that lead to chronic renal injury with subsequent dysfunction, but immune-mediated kidney diseases, such as glomerulonephritis and interstitial nephritis, are also frequent causes of CKD cases (~20%) (2).

In addition to CKD, acute impairment of kidney function (acute kidney injury = AKI) is a common clinical problem that affects up to 25% of hospitalized patients worldwide and represents an important risk factor for in-hospital mortality (3). AKI can result from various clinical conditions, including ischemia, sepsis, and nephrotoxic agents, and usually resolves after successful treatment of the underlying condition or withdrawal of the toxin. However, it has become evident that previous episodes of AKI increase the risk for development of CKD, underlining the importance of AKI for long-term patient outcome (4, 5).

Regardless of the underlying etiology, the local immune response in renal tissue critically contributes to initiation and progression of acute and chronic kidney damage. However, if activated appropriately, regulatory components of the immune system can also promote kidney tissue regeneration and limit renal inflammation (6, 7). Thus, immunomodulatory strategies that are aimed at shifting the balance from a pro-inflammatory, tissue destructive immune response in the kidney to an anti-inflammatory, pro-regenerative response are promising candidates for the development of novel therapies for kidney diseases. In this context, several recent studies identified kidney-residing Innate lymphoid cells (ILCs) as potential therapeutic targets in the attempt to promote tissue regeneration in AKI and/or slow progression of CKD (8).

## Innate Lymphoid Cells

Innate lymphoid cells (ILCs), as a separate family of leukocytes, are considered to represent the innate counterpart of conventional T cells. Similar to T cells, ILCs exhibit lymphoid morphology and produce large amount of cytokines, but in contrast to adaptive lymphocytes, they do not rely on rearranged antigen receptors for activation. Instead, ILCs are equipped with a wide array of receptors to sense, integrate and respond to local cues provided by haematopoietic and non-haematopoietic cells of the tissue niche they reside in.

ILCs are now subdivided into cytotoxic NK cells (or “killer” ILCs) and four groups of “helper” ILCs: ILC1s, ILC2s, ILC3s, and Lymphoid tissue inducer (LTi) cells, based on their expression of specific transcription factor and cytokine profiles, mirroring the classification of CD4^+^ T helper cell subsets into T_H_1, T_H_2, and T_H_17 cells (9–11).

NK cells are the innate cytotoxic counterpart of CD8^+^ T cells, depend on the transcription factors Tbx21 (Tbet) and eomesodermin (Eomes) and produce IFN-γ, granzymes, and perforin after activation. ILC1s resemble T_H_1 cells and, similar to NK cells, express T-bet and IFN-γ, but not Eomes and are less cytotoxic. ILC2s are defined by GATA-3 expression and produce the T_H_2 cytokines IL-13, IL-5, and IL-4, as well as IL-9 and the epidermal growth factor amphiregulin. ILC3s represent the innate T_H_17 counterpart and are characterized by expression of RORγt and AHR, as well as the production of IL-17 and/or IL-22, GM-CSF, and lymphotoxin. Within the ILC3 subset, the expression of Natural cytotoxicity receptors (NCRs, e.g., NKp46, NKp44) further differentiates ILC3s into NCR^+^ and NCR^−^ ILC3s, exhibiting different effector functions (12). Similar to ILC3s, LTi cells, that are essential for the formation of secondary lymphoid organs during embryonic development, depend on RORγt and produce IL-17, IL-22, and lymphotoxin, but recent studies indicate that they develop from a different precursor (11).

In the past decade, helper ILCs were extensively studied and are now recognized as important regulators of immune responses in a variety of organs and inflammatory conditions (13, 14). As largely tissue-resident cells (15), ILCs are adapted to the microenvironment they reside in Ricardo-Gonzalez et al. (16), thus showing organ-specific subset distribution, phenotype, and functional regulation. While the critical function of helper ILCs in barrier organs, such as the intestine, lung, and skin has been elucidated in great detail, knowledge about their tissue-specific properties in the kidney is still emerging. The role of NK cells in kidney health and disease has been recently reviewed elsewhere (17) and will therefore not be discussed here.

## Distribution, Phenotype, and Regulation of Helper ILC Subsets in the Kidney

First evidence that ILC2s represent a major ILC subset in the murine kidney came from a study using IL-5 reporter mice to investigate the distribution of IL-5-expressing ILCs in various tissues. In these analyses up to 7% of all CD45^+^CD90.2^+^ cells were IL-5^+^ non-T cells, representing the kidney-residing ILC2 population (18). A more detailed characterization of the total IL-7Rα (CD127)^+^Lineage^−^ lymphocyte population in the kidney of naïve mice revealed that, depending on the mouse strain (19, 20), ~1–6% of total CD45^+^ lymphocytes are helper ILCs. Among these, IL-5/IL-13-producing GATA-3^+^ ILC2s are indeed the most abundant ILC subset in the kidney (~80%), while RORγt^+^ ILC3s and Tbet^+^Eomes^−^ ILC1s represent only minor fractions (19). Kidney-residing ILC2s share important characteristics with ILC2s in other anatomical locations, such as tissue residency (21) and expression of specific surface receptors that determine their responsiveness to activating and inhibitory stimuli (see below) (19, 20, 22, 23). However, there are first indications of kidney-specific features of the local ILC2 population (24), warranting further investigation.

The healthy human kidney also harbors a CD127^+^CD161^+^Lineage^−^ helper ILC population that accounts for ~0.5% of total lymphocytes. In line with the mouse data, the kidney-residing ILC population in humans contains a considerable percentage of ILC2s (~35%) defined by expression of CRTH2 and the receptors for IL-33 (T1/ST2) and IL-2 (CD25) (19, 21). However, unlike in the mouse, cKit^+^NCR^+^ ILC3s (~15%) and cKit^+^NCR^−^ ILC3s (~40%, possibly containing some ILC precursors (25), are also abundant in the human kidney in non-inflammatory conditions (19).

Strategic positioning of ILCs within barrier tissues is especially important for their function. ILC2s can be detected by immunohistochemical staining in the glomerular and tubulointerstitial compartments of the mouse kidney (19), but it was shown recently that under homeostatic conditions a majority of renal IL-5^+^ ILC2s reside in the perivascular adventitial cuff surrounding the main arterial vessels where they co-localize with kidney dendritic cells (24, 26). Although the functional relevance of this finding for kidney homeostasis is still unclear, it can be speculated that, similar to the lung, stromal adventitial cells provide cytokines, such as IL-33 and TSLP, that might promote ILC2 maintenance in the healthy kidney tissue (27).

## ILCs in Acute Kidney Injury

**Figure 1:**
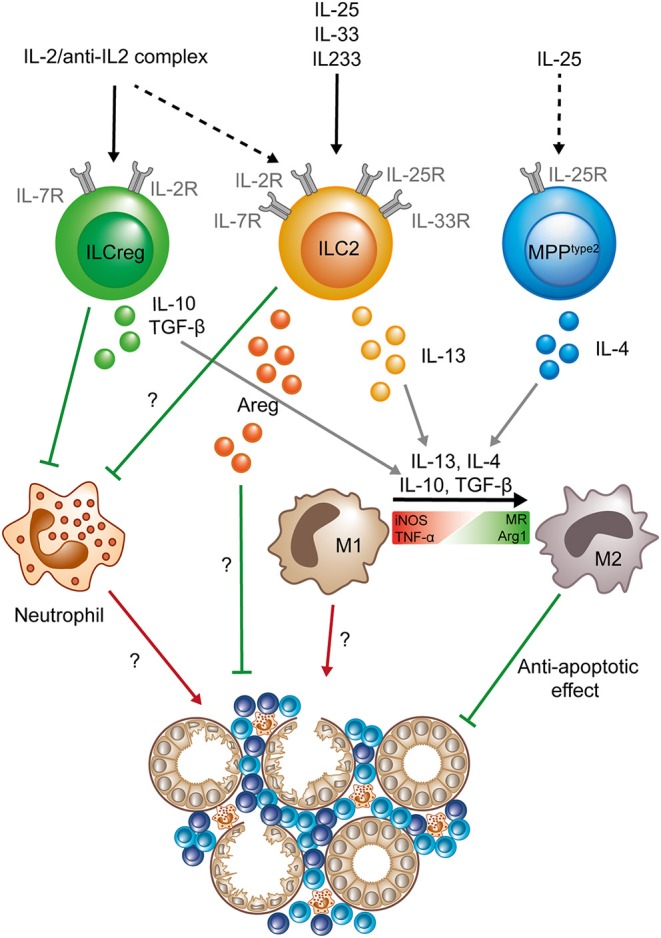
Protective role of ILC2s, MPPtype 2 cells, and “ILCregs” in acute kidney injury. After activation by an IL-2/anti-IL-2 complex (IL2C) ILC2s and “ILCregs” (whether the latter are a separate lineage or IL-10 producing ILC2s is still a matter of debate) prevent neutrophil accumulation in the kidney. “ILCregs” produce IL-10 and TGF-β upon activation. ILC2s can be activated by IL-33, IL-25, the hybrid cytokine IL233, or IL2C and secrete IL-13 and Areg to promote tissue protection. IL-25 can stimulate MPP^type2^ cells to produce IL-4, which in addition to IL-13, IL-10, and TGF-β, has been shown to promote the shift from a pro-inflammatory M1 phenotype (expression of iNOS and TNF-α) to an anti-inflammatory M2 phenotype (expression of MR and Arg1) in macrophages. The exact mechanisms of how ILC2s (and “ILCregs”) prevent neutrophil accumulation and Areg-dependent tissue protection are still unknown. Question marks indicate mechanisms that are so far not completely understood and need to be further elucidated. Green lines symbolize protective and beneficial effects, whereas red arrows indicate proinflammatory effects. (Areg, amphiregulin; Arg1, Arginase 1; iNOS, Inducible nitric oxide synthase; MR, mannose receptor; M1, classical macrophage; M2, alternatively activated macrophage; TNF-α, tumor necrosis factor α; TGF-β, Transforming growth factor β).

Acute kidney injury is characterized by a rapid decrease of kidney excretory function, resulting in elevation of serum creatinine levels and/or decreased urine output (28). Renal ischemia is one major cause of AKI in humans and is induced by various clinical conditions that lead to hypoperfusion of the kidney, such as severe volume depletion, circulatory shock, or renal vascular occlusion. The widely used ischemia/reperfusion injury (IRI) model applies surgical clamping of the renal artery for a defined time period with subsequent reperfusion of the kidney to mimic the pathomechanism of ischemic AKI (29). Similar to ILC2s in other organs, kidney-residing ILC2s express the receptors for IL-25 (IL-17RB) and IL-33 (T1/ST2) and can be activated and expanded *in vivo* by administration of these cytokines in mice (19, 22). Application of ILC2-expanding cytokines has been used to investigate the *in vivo* role of ILC2s in the IRI mouse model of AKI (21, 22). In this model, systemic intraperitoneal application of IL-25 or IL-33 previous to IRI induction resulted in significant renal tissue protection, as indicated by lower serum creatinine levels and reduced tubular damage, accompanied with increased renal expression of the type 2 cytokines IL-4, IL-5, and IL-13 produced by local Lin^−^CD127^+^CD90^+^CD25^+^ST2^+^IL-17RB^+^ ILC2s and, in case of IL-25, by an additional smaller population of Lin^−^CD127^−^CD90^−^ST2^−^CD25^−^IL-17RB^+^c-Kit^+^ Multipotent Progenitor Type 2 Cells (Figure 1). Whether the latter are a separate cell type (30) or represent IL-25-responsive inflammatory ILC2s with low expression of the IL-7 receptor (CD127) (31) remains to be elucidated. The beneficial *in vivo* effects of IL-25 and IL-33 application were indeed mediated by ILC2s, since transfer of IL-25- or IL-33-elicited ILC2s was sufficient to ameliorate renal impairment in mice with IRI (21, 22). Moreover, partial depletion of ILC2s with anti-CD90 antibodies in IL-33-treated *Rag1*^−/−^ mice abolished the protective IL-33 effect, while depletion of Tregs in immunocompetent mice, which have also been described to be IL-33-responsive (32), did not (21). In line with the enhanced intrarenal type 2 response after IL-25 or IL-33 treatment, kidney-residing macrophages were shifted toward a M2 phenotype. Furthermore, neutrophil accumulation in the kidney was reduced by a yet unknown mechanism. The authors could further demonstrate, that *in vitro* differentiated M2 macrophages protected tubular epithelial cells (the primary target cells of ischemic AKI) from apoptosis, providing a potential downstream mechanism for ILC2-mediated tissue protection via alternative activation of macrophages (22). In addition, it was shown that IL-33-activated ILC2s require production of the epidermal growth factor amphiregulin to mediate their protective effects in renal IRI (21), indicating that ILC2s might employ multiple pathways to shift the intrarenal microenvironment from a pro-inflammatory to an anti-inflammatory, pro-regenerative state (Figure 1). Importantly, the therapeutic effect of IL-33 application was maintained when cytokine therapy was started after induction of IRI in mice and was also observed in mice with a humanized immune system that were treated with human recombinant IL-33 (21).

Although these results highlight the therapeutic potential of ILC2-directed therapies in AKI, so far there is no evidence for a role of endogenous ILC2 activation and expansion during AKI. A recent study addressed this issue by comparing tissue injury and renal function impairment between control IRI mice and IRI mice that are reduced or deficient in ILC2s, either constitutively (*Il7r*^cre/+^*Rora*^fl/fl^) or after DTx-mediated depletion (*Cd4*^cre/+^*Icos*^dtr/+^). In these experiments, the authors did not observe a substantial difference in histopathologic tubular injury and inflammatory marker expression in the kidney, leading to the conclusion that endogenous ILC2s that are not previously expanded by cytokine therapy are redundant in IRI (24). Moreover, a previous study provided conflicting evidence for a pro-inflammatory role of IL-33 in AKI by showing that its application in a mouse model of nephrotoxic AKI induced by the cytostatic drug cisplatin aggravates renal injury (33), suggesting that action of IL-33 and IL-33-induced ILC2s, although not specifically addressed in this study, might be highly context-dependent.

A recent study by Cao et al. demonstrated that a small population of IL-10-producing ILCs (2–3% of total ILCs, representing ~0.06% of total lymphocytes) can be detected in the murine and human kidney (34). Definition of these cells was based on a previous report of a similar ILC population in the intestine that was termed “ILCregs” (35), but, since ILC2s can produce large amounts of IL-10 under certain stimulatory conditions (36), it is still a matter of debate if these IL-10^+^ ILCs indeed represent a separate ILC subtype (37). However, the authors went on to show that IL-10-producing ILCs in the kidney can be expanded by IL-2/anti-IL-2 complex (IL2C) treatment and mediate protective effects in the IRI-AKI model by downstream mechanisms similar to IL-25- or IL-33-elicited ILCs (Figure 1) (34), underlining the therapeutic potential of kidney ILCs in AKI.

In the attempt to translate this concept into a therapeutic approach for potential use in human renal disease, a novel hybrid cytokine linking IL-33 with IL-2 has been designed to activate cell types that express a combination of the respective receptors, such as ST2^+^CD25^+^ Tregs and ST2^+^CD25^+^ ILC2s. This hybrid cytokine, termed IL233, was recently shown to be effective in protection from nephrotoxic and IRI-induced AKI by expansion of Tregs and ILC2s (38) and might provide a valuable basis for further development of ILC-directed therapies toward first in-human studies.

## ILCs in Chronic Kidney Disease

**Figure 2:**
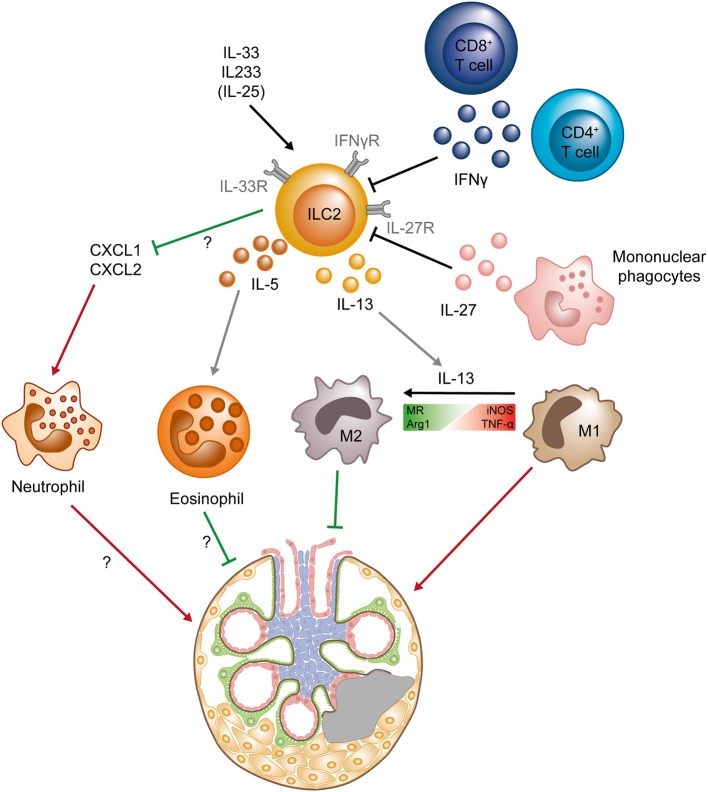
Protective role of ILC2s in chronic kidney diseases. ILC2s can be activated by the cytokines IL-33 and IL-25, as well as the hybrid cytokine IL233, whereas IFNγ (secreted by CD4^+^ and CD8^+^ T cells) and IL-27 (produced by mononuclear phagocytes) suppress ILC2s. Activated ILC2s produce IL-5 and IL-13, leading to the accumulation of eosinophils and the shift from a pro-inflammatory M1 phenotype to an anti-inflammatory M2 phenotype in macrophages. M2 macrophages have been shown to directly protect the tissue, whereas the exact mechanisms of tissue protection mediated by eosinophils are still unclear. The activation and expansion of ILC2s also results in decrease of the chemokines CXCL1 and CXCL2 in the kidney, preventing neutrophil accumulation that mediate renal injury. Question marks indicate mechanisms that are so far not completely understood and need to be further elucidated. Green lines symbolize protective and beneficial effects, whereas red arrows indicate proinflammatory effects. (Arg1, Arginase 1; iNOS, Inducible nitric oxide synthase; MR, mannose receptor; M1, classical macrophage; M2, alternatively activated macrophage; TNF-α, tumor necrosis factor α).

Progressive scarring of the glomeruli (glomerulosclerosis) and fibrosis of the tubulointerstitial compartment are the histopathological hallmarks of CKD. In BALB/c mice, application of the cytostatic drug Adriamycin induces podocyte damage and breakdown of the glomerular filtration barrier, leading to proteinuria, progressive glomerulosclerosis, and chronic tubulointerstitial injury. This “Adriamycin-induced nephropathy” (AN) shares main histopathological features with human CKD and has been widely used as a model to study the effect of therapeutic interventions in proteinuric CKD (39). It was shown previously that, similar to the AKI model, repeated application of IL-25 after AN induction ameliorates its clinical course by induction of M2 macrophages, but the IL-25-responsive cell type responsible for this effect was not addressed in the initial study (40). More recently, our own group showed that a short course of IL-33 treatment in mice (400 ng i.p. on four consecutive days) leads to a massive and sustained increase in kidney ILC2s for up to several month and effectively improved histopathological and clinical parameters of renal injury in the AN model (19). Mechanistically, IL-33-mediated kidney protection in AN was accompanied by an accumulation of eosinophils and a reduction of neutrophil and inflammatory mononuclear phagocyte infiltration. Analysis of ILC-deficient *Rag*^−/−^*Il2rg*^−/−^ mice and eosinophil-deficient ΔdblGATA mice confirmed that the IL-33 effect depended on the presence of ILCs and eosinophils (19) (Figure 2). In line, pre-emptive treatment with the above-mentioned, novel hybrid cytokine IL233 protected mice from progressive glomerulosclerosis in AN (38).

In contrast to the beneficial effects of the IL-33/ILC2 axis in glomerulosclerosis, a potential deleterious role of endogenous IL-33 in kidney fibrosis was reported by Chen et al., demonstrating partial protection from tubulointerstitial fibrosis induced by unilateral urinary obstruction (UUO) in *Il33*^−/−^ and *Il1rl1*^−/−^ mice (41). Accordingly, administration of high-dose IL-33 (500 ng i.p. daily for 14 days) promoted tubulointerstitial fibrosis at week two after IRI-AKI, while inhibition of IL-33 reduced AKI-induced fibrosis. Although the exact cellular mediators and downstream mechanisms of this deleterious IL-33 effect in renal fibrosis were not explored in these studies (41, 42), pro-fibrotic effects of chronically activated ILC2s via production of IL-13 were described in the liver and lung (43, 44), indicating that systemic ILC2-directed therapies might comprise a substantial risk for side effects which are likely to be determined by dose, duration, and context of cytokine application. While higher amounts (1 μg per injection) and/or prolonged application of IL-33 (14 days) might have disadvantageous effects (33, 42), lower doses (0.3–0.5 μg IL-33 or IL-25 per injection) and short-term treatment (3–5 days) were shown to be beneficial (19–22, 34, 40) in various models. Whether systemic ILC2 expansion after i.p. treatment with these cytokines also contributes to the tissue protective effects in the kidney is still unclear and warrants further studies.

Since two independent studies suggested increased numbers of ILC2s and type 2 cytokines (IL-4, IL-5, IL-13) in the peripheral blood of patients suffering from CKD due to type 2 diabetes (45, 46), it can be speculated that ILC2s might be a marker for renal fibrosis in human CKD. However, technical limitations in the flow cytometry gating strategy used to identify ILC2s in these studies preclude valid conclusions from these data and further research is clearly needed to assess a potential role of ILC subsets in human CKD.

## ILCs in Glomerulonephritis

Glomerulonephritides (GNs) are a major cause of CKD and are characterized by a pathogenic immune response against renal autoantigens or by renal manifestations of systemic autoimmune diseases, such as systemic lupus erythematosus (SLE) or anti-neutrophil cytoplasmic antibody (ANCA)-associated small vessel vasculitis. A potential role of ILCs in the pathogenesis of GN is just beginning to be unraveled. In a recent study, our group provided first evidence that kidney-residing ILC2s are decreased in frequency and number with progression of autoimmune renal inflammation in the MRL/MpJ-Fas^lpr^ (MRL-lpr) mouse model of SLE (20). Progression of lupus nephritis in MRL-lpr mice was characterized by marked increase in IFN-γ and IL-27 expression in the inflamed kidneys that were produced by T cells and inflammatory myeloid cells, respectively (20). We and others could further show that, similar to ILC2s in the lung (47, 48), kidney ILC2s express the IFN-γR and IL-27R and are extremely sensitive to IFN-γ/IL-27-mediated inhibition of IL-33-induced proliferation and cytokine production *in vitro* (20, 23), providing a mechanism for inflammation-induced reduction of ILC2s in the kidney (Figure 2). Most importantly, treatment with IL-33 restored kidney ILC2s, increased type 2 cytokine expression and eosinophil accumulation, reduced severity of lupus nephritis, and improved survival of MRL-lpr mice (20), indicating that ILC2s might be protective in immune-mediated glomerular diseases.

While in the MRL-lpr model the other helper ILC subsets were unaltered (20), a recent study suggested that a previously unknown ILC1 subtype expressing CD8 might infiltrate glomeruli in rat and potentially also in human anti-GBM nephritis (49). However, if this CD8^+^ cell subset indeed represents a novel ILC subset needs to be confirmed in future studies.

Initial studies in patients suffering from ANCA-associated vasculitis showed that total ILC numbers in the peripheral blood were reduced in the acute phase of the disease, as compared to healthy controls, which was due to a reduction of both ILC2s and ILC3s (50). Moreover, the authors could demonstrate a significant correlation between a reduction in ILC numbers and high disease activity, supporting the conclusion from the murine SLE model that ILCs might have a protective effect in chronic autoimmunity (20, 50). However, another study analyzing peripheral blood ILC numbers in ANCA vasculitis patients and in appropriate disease controls with a similar impairment of renal function was unable to detect a vasculitis-specific reduction, indicating that a decrease in peripheral ILCs might be a non-specific manifestation of CKD (51).

## Concluding Remarks and Future Directions

In the last decade ILCs have emerged as important effector cells of the innate immune system in a variety of chronic inflammatory and autoimmune conditions. A number of recent studies in preclinical models demonstrate a role of ILC2-directed therapies in promoting kidney regeneration after acute injury and in shifting the intrarenal immune milieu toward a tissue protective type 2 response. However, chronic and systemic over activation of ILC2s might comprise the risk of pro-fibrotic and pro-allergic side effects in the kidney and other organs which have to be considered in the attempt to translate these findings into specific ILC-directed treatment strategies for inflammatory kidney diseases in humans.

So far, there are no comprehensive studies addressing kidney-specific ILC properties, but first data indicate a specific phenotype of the local ILC2 population in the kidney (24). In the future, it will be critical to elucidate the specific molecular pathways that drive kidney ILC activation and to obtain a detailed understanding of their localization and interaction with other immune cells and parenchymal cells within the kidney tissue. These analyses will help to identify pathways that allow for specific targeting of kidney-residing ILCs in the attempt to exploit their tissue protective properties, without causing potential deleterious ILC activation in other anatomical locations.

## Author Contributions

All authors have participated sufficiently in the work to take public responsibility for the content. MB, A-CG, and J-ET drafted, revised, and approved the final version of the manuscript.

### Conflict of Interest

The authors declare that the research was conducted in the absence of any commercial or financial relationships that could be construed as a potential conflict of interest.
